# How children and young people can stay physically active during the novel coronavirus pandemic while take into account safety measures and precautions

**DOI:** 10.34172/hpp.2020.47

**Published:** 2020-11-07

**Authors:** Andreas Fröberg

**Affiliations:** ^1^Department of Food and Nutrition, and Sport Science, University of Gothenburg. Pedagogen, Hus C, Läroverksgatan 5, PO Box 300, SE-405 30, Gothenburg, Sweden

**Keywords:** Adolescent, Child, Coronavirus, Exercise, Quarantin

## Abstract

The novel coronavirus (COVID-19) outbreak has caused major public concern and posed challenges to societies across the globe. The COVID-19 pandemic might have implications for health-related behaviors, such as physical activity, among people in different age groups. Lately, a number of papers have offered suggestions and recommendations on how to stay physically active during the novel coronavirus pandemic while take into account safety measures and precautions. Many of these suggestions and recommendations might be relevant for health professionals and health practitioners working to facilitate physical activity, health, and well-being among children and young people. In light of the COVID-19pandemic, this paper provides an overview of (a) suggestions and recommendations on physical activities; and (b) safety measures and precautions while being physically active.

## Introduction

During the last few months, the novel coronavirus (COVID-19) outbreak has received worldwide attention.^1^ According to the World Health Organization COVID-19 dashboard, more than 20 000 000 confirmed cases of COVID-19, with approximately 800 000 deaths reported as of mid-August 2020.^2^ COVID-19 has reached pandemic levels, caused major public concern and posed challenges to societies worldwide. Therefore, several public health measures have been announced by health organizations and authorities, including social distancing policies and quarantine.

Public health measures, such as social distancing policies and quarantine, are necessary to prevent further spread of the coronavirus but might lead to several negative health consequences. For example, negative psychological effects during quarantines might be post-traumatic stress symptoms, confusion, and anger.^3^ Concern has also been raised that social distancing policies and quarantine might have implications for people’s physical activity behaviour.^4-6^ For example, in the event that public gyms, fitness centers, and public sport arenas have closed, people might have restricted access to several indoor and outdoor environments where they usually are physically active.^7^ In accordance with this concern, one study has shown that worldwide physical activity decreased with more than 25% within 30 days of the COVID-19 pandemic declaration.^8^

Among children and young people, there are compelling evidence suggesting that physical activity is important for health and well-being.^9^ Physical activity might improve not only cardiorespiratory and muscular fitness, cardio-metabolic health, bone health, weight status, and cognition, but also reduce the risk of depression.^9^ The current physical activity recommendations suggest that children and young people aged 6-17 years should engage in 60 min/d or more of moderate-to-vigorous physical activity, of which vigorous physical activity should be included at least three days per week.^9^ Examples of moderate- and vigorous-intensity activities are brisk walking, and jogging and running, respectively.^9^ Children and young people in this age group are also recommended to engage in muscle- strengthening (e.g., resistance training and weight lifting) and bone-strengthening (e.g., running and jump roping) physical activity three days or more per week.^9^

Due to the COVID-19 pandemic, children and young people might engage in less physical activity and accumulate more sedentary behavior, including recreational, passive screen-time (e.g., TV-time), which has been associated with negative health consequences, such as unfavourable body composition and lower fitness.^10^ It should be mentioned that prior to the COVID-19 outbreak, approximately 80% of children and young people aged 11-17 years did not achieve the physical activity recommendations.^11^ Studies published during the last few months indicate that children and young people now have become even less physically active.^12,13^ This is critical as leading health organizations and authorities, such as the American College of Sports Medicine,^14^ have recognized the importance of continue engaging in physical activity during the COVID-19 pandemic.

Lately, a number of papers have offered suggestions and recommendations on how to stay physically active during the novel coronavirus pandemic while take into account safety measures and precautions. Many of these suggestions and recommendations might be relevant for health professionals and health practitioners working to facilitate physical activity, health, and well-being among children and young people. In light of the COVID-19 pandemic, this paper provides an overview of (*a* ) suggestions and recommendations on physical activities; and (*b* ) safety measures and precautions while being physically active.

## Suggestions and recommendations on physical activities

Several papers have recognized the importance of home-based physical activity during the COVID-19 pandemic.^15-44^ Nonetheless, Hammami et al^17^ suggested that those who safely and easily can access outdoor environments, such as parks and fields or similar, are recommended to use these to engage in physical activity. It has also been recognized that parents can be physically active with children, through play and exercise,^16,37^ and that many activities can be performed with family members.^18^

Suggested forms of exercises are aerobic, muscle-strengthening, balance, and stretching (or a combination thereof). Among others, recommended activities are (brisk) walking (outdoor or at home), Nordic walking, jogging, running (outdoor or on the spot at home), bicycling, stair climbing, lifting and carrying groceries or transporting items of moderate weight, as well as household tasks such as cleaning, gardening, washing and ironing clothes, sweeping floors, vacuuming, and mowing the lawn.^15,18,20,22,23,25,27,28,30-32,37-42^ In addition, some papers suggested activities such as Qigong and Yoga, Pilates, and Tai Chi, or dance-based exercise.^15,17,22,23,41^ In their paper, Schwendinger and Pocecco recommended intermittent high-intensity interval training (i.e., alternating bouts of high-intensity exercise with low-intensity recovery periods) with bodyweight exercises, such as push-ups, burpees, air-squats, jumping lunges, single-step climbing, and rope skipping.^19^

The papers also provided several suggestions on specific fitness equipment that can be used when exercising. These included stationary bicycles, rowing ergometers or treadmills, elastic bands and Yoga mats, and weighted arm bands and hand-held weights, such as dumbbells, however the potential benefits of using bodyweight exercises have frequently been acknowledged.^17,23,24,28,39,42,43^ In addition, it has been recognized that everyday household objects can be used as exercise equipment, such as broomsticks, ropes, towels, product packages and (water-filled) bottles, backpacks, books, and furnitures.^18,22,24,26,28,37,39^ Some specific suggestions on exercises were also provided in the papers, such as abdominal crunches, push-ups, plank, squats, split squats, box jumps, burpees, jumping jacks, step-up onto a chair, triceps-dip on a chair, calf raises on the edge of an incline, and jump roping.^15,17,25,26,28,31,37,39,42,43^

Moreover, Margaritis et al^24^ suggested that board games could be modify to incorporate balancing and stretching challenges. In their paper, Calcaterra et al^29^ provided some examples of different game-based physical activities that might be considered. These included, for example, clean-up races (e.g., see who can clean-up their room as quickly as possible), playing with pets, creating obstacle courses (e.g., by means of furniture), and playing follow the leader (i.e., imitate movement patters from the person who assume the role of leader).

Furthermore, a number of papers suggested that health technologies could be used to facilitate physical activity, including videos or smartphone application-guided exercise programs, wearable sensors (e.g., pedometers), online communication, and even phone calls and text messages.^15,21-23,25-28,31-34,36,41,44-48^ For example, Nyenhuis et al^27^ provided a rational for using home-based fitness products to engage in physical activity during homestay. The authors acknowledged the potential of using free-of-charge interactive smartphone fitness applications and easily accessible exercise videos, freely available online via YouTube. In addition, they recognized that several gyms and fitness instructors offer virtual online exercise sessions. Together, these home-based exercise programs might include aerobics, Yoga and Pilates sessions, and muscle-strengthening and stretching exercises, of which some require minimal equipment.^21,23,28^ It has also been acknowledged that active video games might be an appropriate approach to engage in physical activity.^17,18,29,49^ To facilitate motivation and connection to others, Jurak et al^18^ also suggested that it might be useful to upload photographs or videos, representing oneself performing exercise at home, on social media.

As summarized by Rodríguez et al^50^ and Dwyer et al,^33^ several international health organizations and authorities, including American College of Sports Medicine, American Heart Association, Exercise & Sport Science Australia, and the World Health Organization, have provided similar suggestions and recommendations as presented above.

Below is an overview of suggestions and recommendations regarding safety measures and precautions while being physically active during the COVID-19 pandemic.

## Safety measures and precautions while being physically active

In terms of safety measures and precautions, it has, for example, been recommended to pursue safe environments,^23^ maintain social distance,^22,23,40,41,51^ and avoid crowded environments.^20,25,51^ Moreover, Hudson and Sprow emphasized that the highest level of risk for children involves team competitions against peers from other geographic locations.^23^ Halabchi et al^20^ also acknowledged challenges related to different sports activities (e.g., close contact with peers), environments (e.g., pool water) and equipment since these might be risky due to the contagiousness of COVID-19.

Some papers recommended to avoid walking or running directly behind the person ahead, and that side-by-side arrangements should be considered if necessarily.^27,51^ Furthermore, Nyenhuis et al^27^ recommended that 5 and 10 meters social distance should be maintained when brisk walking and running, respectively.

Moreover, Hudson and Sprowemphasize the importance of employing aggressive hygiene and sanitation protocols in indoor environments, such as public gyms and fitness centers.^23^ Others recommended to frequently sanitize exercise equipment (e.g., weight machines, elastic bands, dumbbells and barbells, foam rollers, Yoga blocks, and mats), and avoiding sharing bottles, using cell-phones during exercise in shared indoor environments, as well as not allow children to climb on park equipment, slides, and outdoor fitness equipment, since they might provide a surface for coronavirus transmission.^18,51^

In their paper, Halabchi et al^20^ recognize that moderate physical activity might be beneficial for the immune system with the risk of respiratory track viral infections reduced. Importantly, however, long durations of high-intensity physical activity might weaken the immune system which might increase the risk of inspiratory track infection.^20^

The importance of introducing a beginning exercise program at low intensity and progress gradually, as well as adopting exercises based on physical condition and health status, have been emphasized.^16,19,22,24,28,41^ Furthermore, individuals susceptible to chronic conditions, and those who previously have experienced symptoms of illness, should consult health professionals regarding safe exercise.^15,41^ For safety reasons, exercise should also be interrupted in the event of fever or any signs of COVID-19, such as fatigue and dry cough.^24,30,37^

Furthermore, Jurak et al^18^ recommended to avoid physical activities that might be accompanied with high risk of injury as this might result in over-burdening the public health system and increased risk of exposure of COVID-19.

In their paper, Chen et al^16^ explicitly focused on safety measures and precautions crucial for avoiding a second wave of COVID-19 when children return to the school environment. Similar to above, they recommend proper social distancing, avoid crowding, make hand-washing or hand sanitizer stations easily accessible, regularly sanitize surfaces and equipment, and avoiding sharing bottles and sports equipment. In addition, they suggest to consider activities that maintain social distancing and restricting physical activities that involve body contact. Furthermore, parents were encouraged to practice hygiene with children, such as covering the mouth and nose when coughing and sneezing, avoid touching their eyes, nose, and mouth with unwashed hands. Also, to frequently wash hands, discourage handshakes with peers, and frequently sanitize sport or exercise equipment at home.

## Conclusion

Although physical activities should be selected and adopted based on individual preferences and possibilities, many of the suggestions and recommendations provided in this paper might be relevant for health professionals and health practitioners working to facilitate physical activity, health, and well-being among children and young people. However, caution is required and it is important to undertake safety measures and precautions, such as pursue safe environments, maintain social distance, and avoid crowded environments. In addition, it is important to facilitate hygiene, and interrupt exercise programs in the event of fever or signs of COVID-19.

## Acknowledgements

The author acknowledges the referees for their comments and suggestions.

## Funding

There was no source of funding.

## Competing interests

The author declares no competing interest.

## Ethical approval

Not applicable.

## Author’s contribution

The author conceptualized, designed, reviewed, wrote the draft, and submitted the paper.
